# A simple and rapid protocol for the genetic transformation of *Ensete ventricosum*

**DOI:** 10.1186/s13007-019-0512-y

**Published:** 2019-11-08

**Authors:** Jonathan Matheka, Jaindra Nath Tripathi, Ibsa Merga, Endale Gebre, Leena Tripathi

**Affiliations:** 1 International Institute of Tropical Agriculture (IITA), Nairobi, Kenya; 2 Ethiopian Institute of Agricultural Research (EIAR), Addis Ababa, Ethiopia

**Keywords:** *Ensete ventricosum*, Bedadeti, *Agrobacterium*-mediated transformation, Meristem, Multiple buds, *gfp g*ene

## Abstract

Enset (*Ensete ventricosum*), also known as Ethiopian banana, is a food security crop for more than 20 million people in Ethiopia. As conventional breeding of enset is very challenging, genetic engineering is an alternative option to introduce important traits such as enhanced disease resistance and nutritional value. Genetic transformation and subsequent regeneration of transgenic enset has never been reported mainly due to challenges in developing transformation protocols for this tropical species. *Agrobacterium*-mediated transformation could be a practical tool for the genetic improvement of enset. However, the efficiency of the transformation system depends on several parameters such as plant regeneration, genotype, explant, selection agent and *Agrobacterium* strains. As a first step towards the development of transgenic enset, a simple and rapid plant regeneration system was developed using multiple buds as explants. Induction and proliferation of multiple buds from shoot tip explants was achieved on Murashige and Skoog (MS) medium supplemented with 5 and 10 mg/l of 6-benzylaminopurine (BAP), respectively. Shoots were regenerated from multiple buds on MS media containing 2 mg/l BAP and 0.2% activated charcoal. Based on the optimized regeneration protocol, an *Agrobacterium*-mediated transformation method was developed using multiple buds as explants and the binary plasmid pCAMBIA2300-GFP containing the *green florescent protein (gfp)* reporter gene and *neomycin phosphotransferase* II (*npt*II) selection marker gene. Transgenic plantlets were obtained within 4 months at a frequency of about 1.25%. The transgenic lines were validated by PCR analysis using primers specific to the *npt*II gene. To obtain uniformly transformed plantlets, chimerism was diluted by subculturing and regenerating the transgenic shoots on a selective medium containing kanamycin (150 mg/l) for five cycles. The uniformity of the transgenic plants was confirmed by Southern blot hybridization and RT-PCR analyses on different tissues such as leaf, pseudostem and root of same transgenic plant. In the present study, we report a simple *Agrobacterium*-mediated transformation system for generating transgenic events of enset. To the best of our knowledge, this is the first report on the stable transformation and regeneration of transgenic events of enset. The transformation system established in this study can be used for the generation of transgenic enset with important traits such as disease resistance.

## Background

*Ensete ventricosum* (commonly known as enset or the Ethiopian banana) is domesticated and cultivated in Ethiopia. It is grown on more than 300,000 ha of land in the central and southwestern parts of the country [1]. About 20 million people depend on it for food [2]. It belongs to the family *Musaceae* within the order Zingiberales and genus *Ensete* comprising seven species (*Ensete homblei, E. perrieri, E. ventricosum, E. livingstonianum, E. glaucum, E. superbum* and *E. lecongkietii*). Three of the species (*Ensete ventricosum, E. livingstonianum* and *E. glaucum*) are widespread in Africa and Asia. The ensete species are perennial herbaceous flowering plants. They have been exploited for human food, animal fodder, fiber, construction materials, packaging materials, medicine, ornamental value and alleviation of soil erosion [2–4]. *E. ventricosum* can withstand long periods of drought, heavy rains and flooding. In addition, it can be harvested at any time, even years after the plant reaches maturity, making it an excellent food security crop [4].

The major economically important traits for the genetic improvement of enset include high yielding, taste, nutritious, early maturing and resistance to diseases [5]. Bacterial wilt disease caused by *Xanthomonas campestris* pv. *musacearum* (Xcm) is one of the most important constraints to enset production. The disease was first observed in Ethiopia on enset in the 1930s but was identified as bacterial wilt in the 1960s [6, 7]. The disease is currently prevalent in all the enset-growing regions in Ethiopia. Xcm also adversely affects banana production in East and Central African countries including Uganda, Tanzania, Kenya, Burundi, Rwanda and DR Congo [8, 9].

The application of conventional approaches to enset breeding is greatly hampered by the long reproductive cycles and poor germination of seeds [10, 11]. Genetic engineering techniques may be used to accelerate the incorporation of useful traits into enset. During the last decade, different methods of genetic transformation were developed for several crop species. These include approaches for the direct transfer of nucleic acids into cells with and without carrier particles and *Agrobacterium tumefaciens*-mediated transformation. *Agrobacterium*-mediated transformation as a tool for genetic improvement has been successfully implemented in *Musa* species in programs designed to control fusarium wilt [12, 13], bacterial wilt [14] and nematodes [15]. A major drawback to extending these transgenic approaches to other economically important species in the *Musacae* family (including *E. ventricosum*) is the lack of efficient regeneration protocols [16]. To date, the main tissue culture technique applied to enset is micropropagation using either intercalary meristems [17] or shoot-tips [18–21].

Micropropagation has been successfully exploited in the development of transformation procedures for recalcitrant crops [13, 22–24]. Meristem-based approaches have several advantages over those based on callus or somatic embryogenesis including rapidity and low incidences of somaclonal variations [25]. Unlike somatic embryogenesis, which is limited to only a few cultivars, micropropagation is applicable to a wide range of cultivars irrespective of ploidy or genotype. The main challenge with the meristematic explant-based transformation system is the production of chimeric transgenic plants resulting from gene transfer to a small number of the multiple cells involved in the development of shoot meristem [22, 23]. However, homogeneously transformed plants can be obtained through the manipulation of the tissue culture and selection process [26].

Gene transfer using *Agrobacterium* and regeneration of transgenic plants are influenced by different factors. The key parameters influencing the ability to develop transgenic plants include the type of explant, *Agrobacterium* strain, binary vector, selection agent and compositions of the inoculation and co-culture medium. In addition, treatments such as micro-wounding of explants prior to transformation [27] and vacuum infiltration of explants before co-cultivation with *Agrobacterium* [24] enhanced the transformation of different plant species. Currently, there are no reports of stable genetic transformation and regeneration of transgenic events of enset. Birmeta [19] provided the evidence showing that enset tissues are sensitive to *Agrobacterium* infection, however, regeneration of transgenic plants from the transformed tissues and stable integration of transgene were not demonstrated. In the report, zygotic embryos, leaf and root tissues of two clones of enset (Feresae and Erba) were transiently transformed with the *gusA* reporter gene.

In the present study, we report the development of a simple and rapid protocol for the stable transformation and regeneration of transgenic events of enset using multiple meristematic buds. To significantly improve the transformation efficiency, we optimized the effect of micro-wounding through sonication, different *Agrobacterium* strains, co-cultivation media and resting duration. The effect of these parameters was studied individually and then we came up with a comprehensive protocol where all these modifications were combined to attain maximum transformation efficiency. In addition, we have developed a protocol for the elimination of chimeras in transgenic plantlets. Using this protocol, we obtained uniformly transformed plantlets, thus overcoming the main obstacle in the genetic manipulation of enset.

## Materials and methods

### Plant materials

*Ensete ventricosum* cv. Bedadeti used in this study was acquired from the Ethiopian Institute of Agricultural Research (EIAR), Ethiopia, as in vitro plantlets. The plantlets were multiplied by monthly subculturing on shoot elongation medium (SEM4, Table 1) under controlled temperature (25 ± 2 °C) and light (16:8-h photoperiod and 80 mmol/m^2^/s irradiance) conditions in a growth room.

**Table 1:** Composition of various media used in tissue culture and transformation of enset

Medium name	Components
Bud induction medium 1 (BIM1)	MS^a^ medium supplemented with 10 mg/l ascorbic acid, 30 g/l sucrose and 2.5 mg/l BAP, pH5.8 and 3 g/l gelrite
Bud induction medium 2 (BIM2)	MS medium supplemented with 10 mg/l ascorbic acid, 30 g/l sucrose and 5 mg/l BAP, pH5.8 and 3 g/l gelrite
Bud induction medium 3 (BIM3)	MS medium supplemented with 10 mg/l ascorbic acid, 30 g/l sucrose and 10 mg/l BAP, pH5.8 and 3 g/l gelrite
Bud induction medium 4 (BIM4)	MS medium supplemented with 10 mg/l ascorbic acid, 30 g/l sucrose and 15 mg/l BAP, pH5.8 and 3 g/l gelrite
Bud Induction Medium 5 (BIM5)	MS medium supplemented with 10 mg/l ascorbic acid, 30 g/l sucrose and 22 mg/l BAP, pH5.8 and 3 g/l gelrite
Bud multiplication medium 1 (BMM1)	MS medium supplemented with 10 mg/l ascorbic acid, 30 g/l sucrose, 0.175 mg/l IAA, 2.5 mg/l BAP, pH5.8 and 3 g/l gelrite
Bud multiplication medium 2 (BMM2)	MS medium supplemented with 10 mg/l ascorbic acid, 30 g/l sucrose, 0.175 mg/l IAA, 5 mg/l BAP, pH5.8 and 3 g/l gelrite
Bud multiplication medium 3 (BMM3)	MS medium supplemented with 10 mg/l ascorbic acid, 30 g/l sucrose, 0.175 mg/l IAA, 10 mg/l BAP, pH5.8 and 3 g/l gelrite
Bud multiplication medium 4 (BMM4)	MS medium supplemented with 10 mg/l ascorbic acid, 30 g/l sucrose, 0.175 mg/l IAA, 15 mg/l BAP, pH5.8 and 3 g/l gelrite
Bud multiplication medium 5 (BMM5)	MS medium supplemented with 10 mg/l ascorbic acid, 30 g/l sucrose, 0.175 mg/l IAA, 22 mg/l BAP, pH5.8 and 3 g/l gelrite
Shoot elongation medium 1 (SEM1)	MS medium supplemented with 10 mg/l ascorbic acid, 30 g/l sucrose, 0.2% AC, 0.1 mg/l BAP, pH5.8 and 3 g/l gelrite
Shoot elongation medium 2 (SEM2)	MS medium supplemented with 10 mg/l ascorbic acid, 30 g/l sucrose, 0.2% AC, 0.5 mg/l BAP, pH5.8 and 3 g/l gelrite
Shoot elongation medium 3 (SEM3)	MS medium supplemented with 10 mg/l ascorbic acid, 30 g/l sucrose, 0.2% AC, 1.0 mg/l BAP, pH5.8 and 3 g/l gelrite
Shoot elongation medium 4 (SEM4)	MS medium supplemented with 10 mg/l ascorbic acid, 30 g/l sucrose, 0.2% AC, 2.0 mg/l BAP, pH5.8 and 3 g/l gelrite
Shoot elongation medium 5 (SEM5)	MS^a^ medium supplemented with 10 mg/l ascorbic acid, 30 g/l sucrose, 0.2% AC, 3.0 mg/l BAP, pH5.8 and 3 g/l gelrite
Root induction medium 1 (RIM1)	MS medium supplemented with 10 mg/l ascorbic acid, 30 g/l sucrose, 1 mg/l IBA, pH5.8 and 3 g/l gelrite
Root induction medium 2 (RIM2)	MS medium supplemented with 10 mg/l ascorbic acid, 30 g/l sucrose, 1 mg/l IBA, 0.1 mg/l BAP, pH5.8 and 3 g/l gelrite
Root induction medium 3 (RIM3)	MS medium supplemented with 10 mg/l ascorbic acid, 30 g/l sucrose, 1 m g/l IBA, 0.25 mg/l BAP, pH5.8 and 3 g/l gelrite
Root induction medium 4 (RIM4)	MS medium supplemented with 10 mg/l ascorbic acid, 30 g/l sucrose, 1 m g/l IBA, 0.5 mg/l BAP, pH5.8 and 3 g/l gelrite
Cocultivation medium A (CCA)	MS medium supplemented with 30 g/l sucrose, 10 mg/l Ascorbic Acid, 1 mg/l BAP, pH 5.8 and 0.3% gelrite. Acetosyringone (200 µM) was added post-autoclaving
Cocultivation medium B (CCB)	MS medium supplemented with 30 g/l sucrose, 10 mg/l Ascorbic Acid, 1 mg/l BAP, 10 g/l glucose, pH 5.8 and 0.3% gelrite. Acetosyringone (200 µM) was added post-autoclaving
Regeneration medium (RM)	MS medium supplemented with 10 mg/l ascorbic acid, 1 mg/l BAP, 30 g/l sucrose, pH5.8 and 3 g/l gelrite
Infection medium (IM)	MS salts (M0222) supplemented with 10 mg/l ascorbic acid, 1 mg/l BAP, 30 g/l sucrose, pH 5.8, and 3 g/l gelrite and 200 µM acetosyringone added after autoclaving
Resting medium (RestM)	MS salts (M0222) supplemented with 10 mg/l ascorbic acid, 1 mg/l BAP, 30 g/l sucrose, pH 5.8, and 3 g/l gelrite and 300 mg/l cefotaxim added after autoclaving
Selective regeneration medium (SRM)	MS salts (M0222) supplemented with 10 mg/l ascorbic acid, 1 mg/l BAP, 30 g/l sucrose, pH 5.8, and 3 g/l gelrite and 300 mg/l cefotaxim and 150 mg/l kanamycin added after autoclaving

*BAP* 6-benzylaminopurine, *IAA* indole acetic acid, *2,4*-*D* 2,4-dichlorophenoxyacetic acid, *Ac* activated charcoal

^a^Murashige and Skoog [45]

### Optimization of enset regeneration using multiple buds as explants

**Fig. 1:**
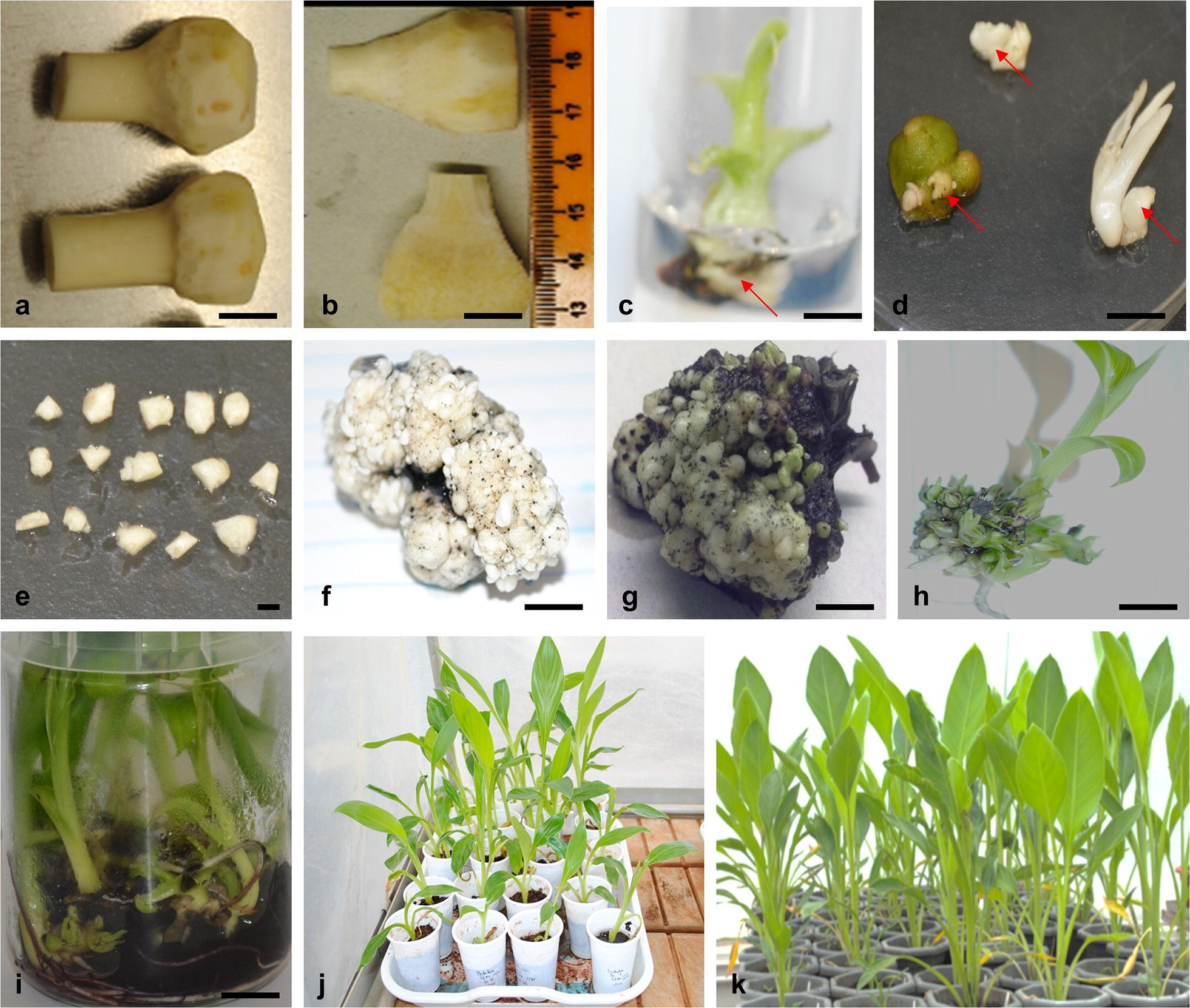
Regeneration of enset through multiple buds. **a** Shoot tip, **b** half split shoot tips used as explants for bud induction, **c** bud induced (indicated by arrow) from shoot tip explants cultured on the bud induction medium (BIM), **d** explants showing induced buds (indicated by arrow), **e** thin slices of excised buds induced on the shoot tips cultured on BIM, **f** multiple buds induced on explant cultured on the bud multiple medium (BMM), **g** induction of sprouting of buds on the shoot elongation medium (SEM), **h** shoots regenerated from multiple buds cultured on SEM having 2.0 mg/l BAP and 0.2% activated charcoal, **i** complete plantlets on SEM, **j** enset plantlets undergoing weaning in a humidity chamber in the glasshouse, **k** potted enset plants growing in the glasshouse. Scale bar = 1 cm for **a**–**d**, **f**–**i** and 2 mm for **e**

The effect of different concentrations (2.5, 5, 10, 15, 22 mg/l) of 6-Benzylaminopurine (BAP) on the induction of multiple buds was evaluated. Shoot tips having a small section of corm tissue with some leaf primordia (Fig. 1a) were isolated from in vitro plantlets by removing the roots and pseudostem. The shoot tip was split longitudinally into two (Fig. 1b) and cultured on bud induction medium (BIM) containing different concentrations of BAP (Table 1). Explants were kept in a growth room at 25 °C in the dark for 6 weeks. The explants were transferred to a fresh medium every 3 weeks and observed for bud induction (Fig. 1c, d). The weight of each explant was recorded before culture and after 6 weeks of culture. There were 6 replicates for each treatment, and the experiment was repeated twice.

The effect of different concentrations (2.5, 5, 10, 15, 22 mg/l) of BAP was evaluated for the proliferation of induced multiple buds. A thin dorm-shaped slice (2 mm thick, 3–4 mm long and 2–3 mm wide) (Fig. 1e) was excised from the buds induced on the shoot tips cultured on BIM2 containing 5 mg/l BAP. The bud explants were cultured on bud multiplication medium (BMM) containing various concentration (2.5, 5, 10, 15, 22 mg/l) of BAP and 0.175 mg/l IAA (Table 1). Explants were kept in a growth room at 25 °C in the dark for 8 weeks. The cultures were subcultured to fresh medium every 4 weeks for bud proliferation (Fig. 1f, g). The weight of each proliferating bud clump was recorded after 8 weeks of culture. There were 4 replicates for each treatment, and the experiment was repeated twice.

The effect of different concentrations (0.1, 0.5, 1.0, 2.0, 3.0 mg/l) of BAP on shoot regeneration from multiple buds was evaluated. Dorm-shaped explants (2 mm thick, 3–4 mm long and 2–3 mm wide) having multiple buds were excised from 90-day-old cultures proliferating on BMM3 and transferred to shoot induction and elongation media (SEM) containing different concentrations of BAP (Table 1). The cultures were incubated at a photoperiod of 16 h light/8 h dark at 25 °C for 8 weeks with subculturing to fresh medium every 4 weeks. The percentage of explants inducing shoots, number of shoots per explant and shoot length were recorded after 8 weeks of culture. Three explants were used for treatment in each medium and the experiment was repeated three times.

To evaluate the rooting response of shoots, media supplemented with 1 mg/l IBA and different concentrations (0.1, 0.25, 0.5, 1.0, 2.0 mg/l) of BAP were tested. Shoots (1.0–1.5 cm long) were excised from regenerating bud clumps (Fig. 1h) and cultured on root induction media (RIM) containing IBA and different concentrations of BAP (Table 1). Three explants were used for each media treatment and replicated three times. Cultures were incubated at 25 ± 2 °C with a 16/8 h photoperiod. Data on shoot length, number of roots per shoot and length of roots per explant was recorded after 7 days.

Complete plantlets were maintained on SEM with 2 mg/l BAP and 0.2% activated charcoal (Fig. 1i). Well-rooted plantlets were removed from the culture media and gently washed with water to remove any remaining medium from the roots. The clean plantlets were then transferred to plastic disposable cups containing sterile soil (80% loam soil and 20% manure) (Fig. 1j). Plantlets were placed in a high humidity chamber (made of transparent polythene sheets) in the glasshouse and watered with tap water. Plantlets were removed from the chamber after 2 weeks and transferred to pots (10 l) containing sterile soil (Fig. 1k). Plants were maintained under the humid chamber in the glasshouse. Data on survival of plantlets in the pots and number of plants showing somaclonal variations was recorded 4 weeks after potting.

### Optimization of *Agrobacterium*-mediated transformation of enset using multiple buds as explants

#### Kill curve analysis of multiple buds on medium containing kanamycin

The sensitivity of multiple buds to kanamycin was tested by culturing the explants on RM (Table 1) supplemented with various concentrations of kanamycin (0, 50, 100, 150, 200 mg/l). The cultures were incubated at 25 ± 2 °C with a 16/8 h photoperiod. The tissues were assessed for shoot regeneration up to 60 days after culture. This assay was performed to establish the minimum inhibitory concentration for the selection of transformed tissues. A positive control in which explants were cultured without a selection agent was included.

#### Bacterial strains and plasmid constructs

*Agrobacterium tumefaciens* strains EHA105 or LBA4404 harboring the binary vector pCAMBIA2300-GFP (Additional file 1: Fig. S1) was used in this study. The vector contains a *green fluorescent protein* (*gfp*) reporter gene under the control of a constitutive CaMV35S promoter. The plasmid vector also contains the *neomycin phosphotransferase II* (*npt*II) gene under the regulation of a CaMV35S promoter for plant selection. EHA105 is a super virulent strain of the agropine type having the pEHA105 helper plasmid [28], while LBA4404 is an octopine type strain having the pAL4404 helper plasmid [29]. *A. tumefaciens* LBA4404:pCAMBIA2300-GFP or EHA105:pCAMBIA2300-GFP cells were streaked on solid Luria–Bertani (LB) medium (1% tryptone, 0.5% yeast extract, and 1% NaC1, pH 7.0) containing 50 mg/l kanamycin and 25 mg/l rifampicin. The cultures were incubated in the dark at 28 °C for 48 h. One colony was selected and inoculated in liquid LB medium. The cultures were kept in the dark at 28 °C with shaking (New Brunswick Scientific incubator shaker, Model Innova 44, Eppendorf AG, Germany) at 200 revolutions per minute (rpm) for 48 h. The *Agrobacterium* culture was used as a seed culture to inoculate 100 ml of LB medium and cultured for 12 h at 28 °C. The culture was centrifuged for 10 min at 4000 rpm and resuspended in 25 ml of infection media (IM) (Table 1). The culture was incubated at 28 °C with shaking at 60 rpm for 1 h. OD_600_ of the culture was adjusted to 0.5 before co-cultivation with explants.

#### Expression of *gfp* reporter gene in transgenic tissues

Tissues from transgenic and control non-transgenic lines were examined under a fluorescence stereomicroscope (SMZ 1500, Nikon Corporation, Tokyo, Japan) at an excitation B filter for wavelength ranges between 460 and 490 nm. Chlorophyll was partially removed from leaf tissues obtained from putative transgenic and wild type plants before examination. This was achieved by treating the leaf tissues with methanol for 20 min followed by soaking in distilled water. Photographs were taken using a digital camera attached to the stereomicroscope.

#### Effect of co-cultivation media on transformation efficiency

The effect of co-culture media on enset genetic transformation was evaluated. Multiple buds (2 mm thick, 3–4 mm long and 2–3 mm wide) isolated from 3-month old proliferating multiple bud cultures were collected into 50 ml tubes containing about 30 ml of infection medium (Table 1). The multiple buds were infected with EHA105:pCAMBIA2300-GFP for 30 min and vacuum infiltrated for 5 min. The *Agro*-infected tissues were co-cultivated for 3 days on either co-cultivation medium A or B (CCA or CCB, Table 1). For each medium treatment, 30 explants were used. UV illumination from a fluorescence stereomicroscope was used to detect *gfp* fluorescence on the explants after 3 days. Data on number of explants with *gfp* fluorescence were recorded.

#### Effect of sonication on transformation efficiency

To determine the effect of sonication on the genetic transformation of enset, explants isolated from 3-month old proliferating multiple buds were sonicated (Decon FS400b, 400 W, 35–45 kHz, Decon LTD., UK) for different durations [0, 5, 50, or 100 s (sec)]. Sonicated explants were infected with EHA105:pCAMBIA2300-GFP and cultured on CCA for 3 days in the dark at 25 °C. The expression of *gfp* gene was determined as green fluorescence for each infected explant. Each experiment comprised 30 explants with three replicates each. Data on number of explants with *gfp* fluorescence were recorded 3 days after co-cultivation.

#### Effect of *Agrobacterium* strain on transformation efficiency

To establish the effect of *Agrobacterium* strains on enset transformation, explants from 3-month old multiple buds were infected with EHA105:pCAMBIA2300-GFP or LBA4404:pCAMBIA2300-GFP. The explants were cultured in CCA and kept in the dark at 22 °C. Data on the number of explants with *gfp* fluorescence were recorded 3 days after co-cultivation. Each experiment comprised 30 explants with three replicates.

#### Effect of resting duration on shoot bud regeneration from Agro-infected explants

To evaluate the effect of resting on enset transformation, explants from 3-month old proliferating multiple buds were infected with EHA105:pCAMBIA2300-GFP and cultured in CCA. Explants were kept in the dark at 22 °C for 3 days. Explants were washed by transferring 50 explants to 50 ml sterile plastic tubes with liquid CM media having 500 mg/l cefotaxime. Explants were incubated at room temperature with shaking (70 rpm) on an orbital shaker. Washed explants were blotted dry and cultured on resting media (RestM) for different durations (0 or 30 days) before transferring to selective regeneration media (SRM) containing 150 mg/l kanamycin. The number of kanamycin-resistant explants with sprouting buds was recorded 30 days after transfer of explants to SRM.

#### Stable transformation of enset using multiple buds as explants

A total of 400 explants excised from 90-day old cultures of multiple buds were kept in eight 50 ml tubes each containing 30 ml of infection media (Table 1). The explants were sonicated for 5 s and then co-cultivated with LBA4404:pCAMBIA2300-GFP suspension (OD600 = 0.5) at room temperature (25 °C) with shaking at 30 rpm for 30 min. The bacterial cell suspension was prepared as previously described. An experiment in which sonicated explants were not infected with bacteria was included as the control. Tissues were blotted dry, cultured on CCA media (Table 1) and incubated at 22 °C for 3 days. Explants were placed on regeneration medium (RM) without selection (Table 1) for 7 days at 26 °C in the dark for resting. After that, explants were transferred to SRM containing 150 mg/l kanamycin and 300 mg/l cefotaxime for 21 days in the dark. All tissues were subcultured twice in the fresh SRM. The control non-transformed explants were also cultured on selective medium. The putatively transformed shoots were regenerated on SRM. Meristem, leaf and root tissues were excised from the putatively transformed shoots regenerated on SRM to confirm the stable expression of *gfp* gene. To obtain uniformly transformed plants, all putative transgenic shoots were treated to a further round of selection on 150 mg/l kanamycin. To achieve this, shoot tip explants (1 cm long × 0.5 cm wide) from putative transgenic plantlets were cultured on RIM3 with 150 mg/l kanamycin (named as CDM1 media) for shoot elongation. After 7 days of culture, explants were transferred to RIM1 with 150 mg/l kanamycin (named as CDM2 media) for 2 weeks for root development. This process was repeated five times. Plantlets having a robust rooting system and fully developed green leaves were deemed non-chimeric, uniformly transformed and further confirmed through molecular analyses. Confirmed transgenic plantlets were maintained on SEM4 medium.

### Molecular analyses

#### Polymerase chain reaction (*PCR) analysis*

PCR analysis was performed to confirm the presence of transgene in the GFP-fluorescing transgenic plants. Genomic DNA (gDNA) was isolated from young leaf, pseudostem or roots of putative transgenic and control non-transgenic plants using the DNeasy kit (Qiagen, GmbH, Germany). The gDNA was subjected to PCR analysis to detect the presence of *npt*II gene with a predicted fragment size of 780 bp. Plasmid pCAMBIA2300-GFP and gDNA from a non-transgenic plant were used as a positive and negative control, respectively. Specific primers for *npt*II gene used were: forward 5′ GATGGATTGCACGCAGGTTCTC 3′ and reverse 5′ CAGAAGAACTCGTCAAGAAGGC 3′. PCR was carried out in a 25 µl reaction volume containing (1X) HotStart master mix (Qiagen) and 100 ng of sample DNA. The reaction mixtures were subjected to the following amplification conditions: initial denaturation at 95 °C for 15 min, 25 cycles of denaturation at 94 °C for 30 s, annealing at 68 °C for 30 s, extension at 72 °C for 1 min and a final extension at 72 °C for 2 min. The amplified fragments were separated by electrophoresis on 1% (w/v) agarose stained with gel red. In total, 12 putative transgenic events were tested by PCR to confirm the presence of *npt*II gene.

#### Southern blot analysis

For additional confirmation of transgene integration, Southern blot analysis was performed with two PCR positive events using the method described in [14]. Briefly, gDNA was extracted from leaf tissues using the CTAB extraction method. Twenty micrograms of gDNA were digested overnight at 37 °C with HindIII, which cuts only once within the T-DNA region of the plasmid vectors used in transformation. Digested DNA was separated by electrophoresis in 0.8% agarose gel. The DNA was blotted onto a positively charged nylon membrane (Roche Diagnostics, Lewes, East Sussex, UK) by upward capillary in a 20× SSC buffer. A digoxigenin (DIG)-dUTP labelled *npt*II probe was hybridized to the blotted DNA. Hybridizing bands corresponding to the *npt*II gene were visualized by exposure to an X-ray film (Roche Diagnostics, Lewes, East Sussex, UK) for 30 min.

#### Reverse transcription polymerase chain reaction (RT-PCR) analysis

RT-PCR was performed to confirm transgene expression on uniformly transformed plants. Total RNA was extracted from leaf, pseudostem and roots of two transgenic events using the RNeasy plant mini kit (Qiagen, GmbH, Germany). DNase I treatment was performed for all samples. For each sample, 1 µg RNA was used for cDNA preparation using the ProtoScript First strand cDNA synthesis kit (New England Biolabs Inc., Ma, USA). Two microliters of cDNA were used in a PCR reaction to detect *npt*II gene transcripts using gene-specific primers. For each sample cDNA, primers for the endogenous small subunit rRNA gene were used for internal control amplification.

### Data collection and statistical analysis

All experiments were conducted as completely randomized designs. Data on frequency of GFP positive explants, bud induction frequency, number of shoots per explant, shoot height, root length and number of roots were collected. Data on frequency of GFP positive explants and bud induction frequency were square root transformed before analysis. Data were subjected to analysis of variance and treatment means were compared using Tukey’s test (*p *= 0.05) and R statistical programming language.

## Results and discussion

This study was conducted to establish an efficient *Agrobacterium*-mediated transformation system for enset cultivar Bedadeti. This system could then be applied to other farmer-preferred enset cultivars. We tried to transform enset using multiple meristematic buds as explants since this approach is potentially rapid and applicable to different cultivars. We then optimized the regeneration of Bedadeti using multiple buds as explants to make the procedure more efficient. The optimized regeneration protocol was adapted to transformation.

### Optimization of enset regeneration using multiple buds

#### Induction and proliferation of multiple buds

**Fig. 2:**
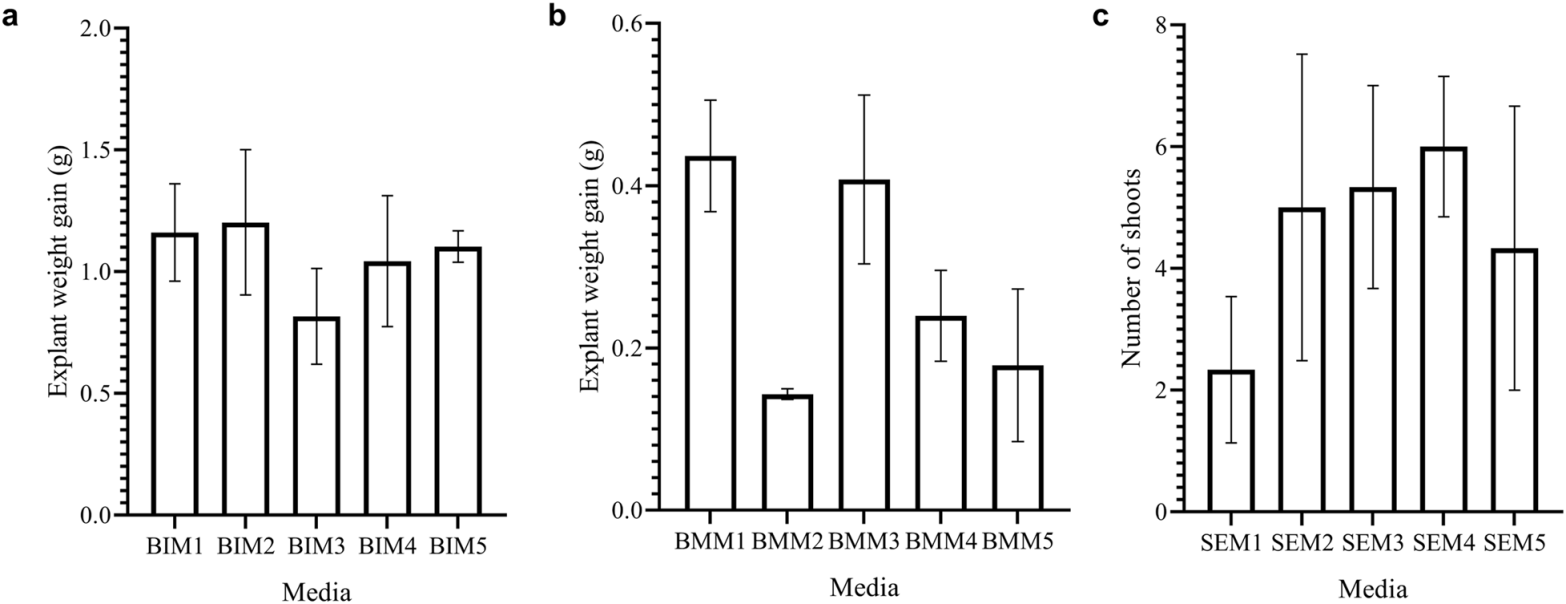
Effect of different media on bud induction, bud proliferation and shoot regeneration from multiple buds of enset. **a** Effect of different media on weight gain of shoot tip explants cultured on bud induction medium (BIM), **b** effect of different media on weight gain of explants cultured on bud multiplication medium (BMM), **c** effect of different media on the number of shoots regenerated from cluster of multiple buds cultured on shoot elongation medium (SEM). The bars represent mean and standard error for values of weight gain or number of shoots

Media containing different concentrations of BAP were tested for efficiency of bud induction from shoot tip explants. Four weeks after culture, buds as smooth white swellings were induced on the explants cultured on all the media tested (Fig. 1c, d). The response of bud induction from shoot tip explants slightly varied according to the amount of BAP used in the proliferation media. However, no significant differences (*p *= 0.7570) were observed among the different media tested for bud induction. The BIM1 medium promoted bud induction from shoot tip explants after 30 days of culture. However, the buds sprouted quickly upon sub-culturing on the same medium, making the medium unsuitable for sustained bud proliferation. Multiple buds were most efficiently induced on shoot tips in the presence of media with 5 mg/l BAP (BIM2), which resulted in a 1.2 g weight gain per explant after 6 weeks of culture (Fig. 2a). Higher levels of BAP (10 or 22 mg/l) induced buds that grew slowly and were highly blackened. Based on these observations, explants were cultured on media supplemented with 5 mg/l BAP (designated as bud induction medium [BIM] for further experiments) for optimal bud induction. Previous studies reported bud induction at lower BAP concentrations (1.5–4.5 mg/l) [18, 19] or in combination with an auxin such as IAA or ABA [19, 20].

To obtain the best response for bud proliferation, several media with different concentrations of BAP and 0.175 mg/l IAA were compared. The growth response of explants cultured on the different media is shown in Fig. 2b. The best growth response was obtained with media treatment BMM1 (with 2.5 mg/l BAP) and BMM3 (with 10 mg/l BAP). However, the optimal media treatment was BMM3, which resulted in bud proliferation (Fig. 1f) with relatively lower regeneration than treatment BMM1 which resulted in sprouting of multiple buds 14 days after culture of explants. Cultures on BMM4 (15 mg/l BAP) and BMM5 (22 mg/l BAP) were either unresponsive or grew very slowly due to intense blackening. Therefore, the medium with 10 mg/l BAP (designated as bud multiplication medium [BMM]) was used for bud multiplication in subsequent experiments.

For enset, media with a high cytokinin/auxin ratio is commonly used for bud multiplication. A 2.5–20-fold higher cytokinin than auxin concentration was essential for multiplication of buds for some enset cultivars [19, 21, 30, 31]. However, in this study, bud multiplication was achieved for enset cultivar Bedadeti with a 50-fold higher BAP than IAA concentration. Growth conditions for bud multiplication include temperatures of 23–27 °C and a 16-h photoperiod at 3000 lx [20, 30, 31]. However, this study reports that buds are best multiplied in the dark to minimize sprouting and blackening. Incubation of explants at shortened illumination duration or in complete darkness has been used to reduce explant darkening in banana [32, 33]. Black coloration of in vitro cultured explants has been attributed to the accumulation of oxidized phenolics in cells resulting from the action of polyphenol oxidases on phenolic compounds exuded from stressed tissues [34]. Oxidized phenolic compounds are highly phytotoxic leading to cell death and release of phenolic compounds in growth medium [35–37]. Maintenance of plant tissue cultures in the dark decreases the activity of enzymes involved in the biosynthesis and oxidation of phenolic compounds [38].

#### Regeneration of complete plantlets

The regeneration of shoots from multiple buds was tested on several SEM supplemented with different BAP concentrations and containing activated charcoal. Shoots started sprouting on all the media as early as 10 days after culture of multiple buds (Fig. 1g). After 60 days of culture, most shoots achieved a length of at least 2 cm (Fig. 1h). The highest number (6.0) of shoots per cluster of multiple buds was obtained on SEM4 containing 2 mg/l BAP media as compared to SEM2 with 0.5 mg/l BAP (5.0) and SEM3 with 1.0 mg/l BAP (5.3) (Fig. 2c). However analysis of variance indicated no significant difference among media treatments in the number of axillary shoots per explant. Our results agree with previous reports of successful shoot regeneration using media containing either BAP alone or a combination of high BAP with low auxin. Genene and Mekbib [18] reported the development of 20–23 shoots per explant per subculture for different enset cultivars cultured on media having 4.5–6 mg/l BAP and 2 mg/l NAA. In this study SEM4 media with 2 mg/l BAP (hereafter referred to as SEM) was identified as the most ideal for shoot regeneration and elongation. The SEM medium is used routinely for shoot regeneration and plantlet maintenance (Fig. 1i).

**Fig. 3:**
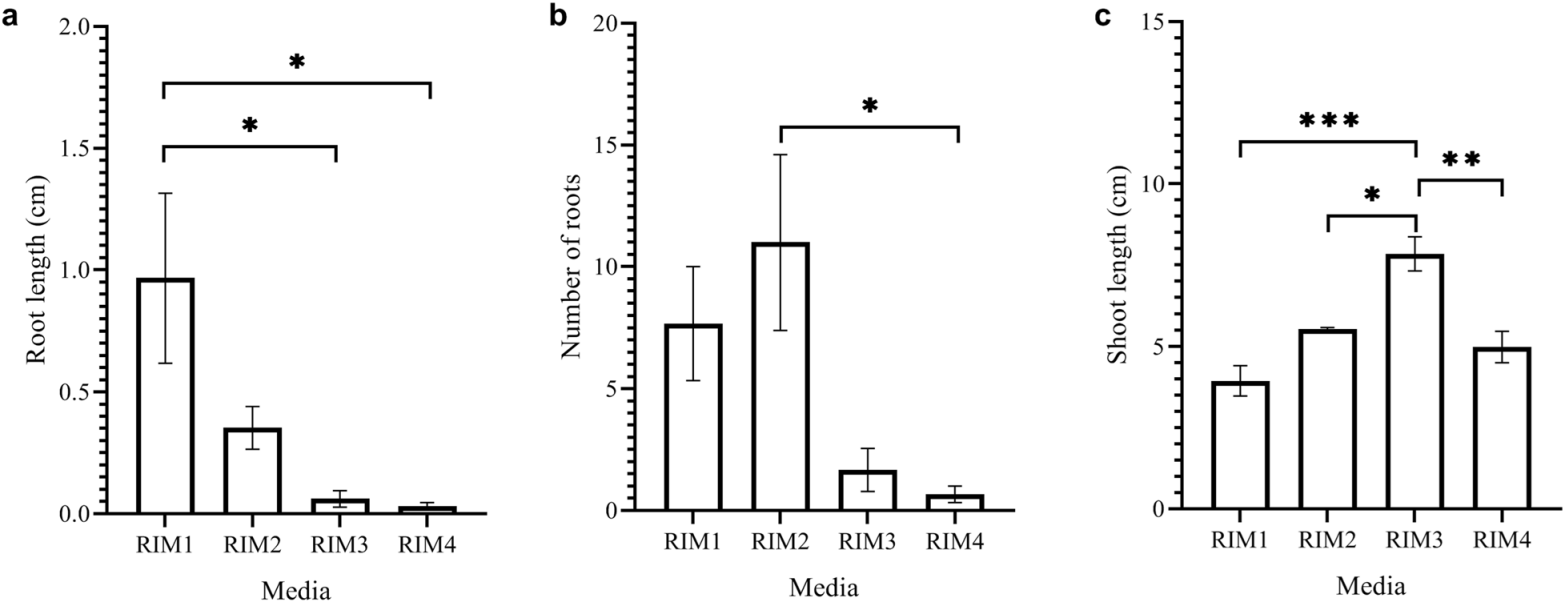
Effect of different media on root and shoot development from multiple buds. **a** Effect of different media on root length, **b** effect of different media on number of roots,** c** effect of different media on shoot length. The bars represent mean and standard error for values on root length, number of roots or shoot length. Asterisk (*) indicate significant difference between means at *p *< 0.05

Shoots were tested on different media for their ability to grow and initiate roots. Root development occurred 3–5 days after culture of shoots on all media. Root length varied from 0.03 to 0.97 cm across the tested media. There was no significant difference (*p* > 0.05) between RIM1 and RIM2 in root length. However, RIM1 treatment rendered significantly higher values (*p* < 0.05) than RIM3 and RIM4 in root length (Fig. 3a). This indicated that RIM1 was the most effective media for root induction and growth. Although roots can be induced on enset shoots using hormone-free media [19], the rooting frequency can be increased by the inclusion of between 1 and 2 mg/l IBA [19, 21]. In this study, roots were induced as early as 3 days after culture of shoots on rooting medium. A maximum of 12 roots per shoot were obtained on RIM2 media with 1 mg/l IBA and 0.1 mg/l BAP (Fig. 3b). Previous studies have reported the induction of a fewer number of roots within a longer culture duration. Genene and Mekbib [18] reported the production of an average of 3.55 roots per explant after 12–14 days post culture of explants on MS media with 1 mg/l IBA. This differences in root induction response can be explained by the fact that root development is dependent on several factors including media type and plant genotype. While root development decreased with increasing concentration of BAP in the culture media (Fig. 3a–b), shoot development increased with increasing BAP concentration (Fig. 3c). The longest shoots were obtained on RIM3 media (Fig. 3c). RIM3 treatment rendered significantly higher values than RIM1 (*p* = 0.0009), RIM2 (*p* = 0.0211) and RIM4 (*p* = 0.0065) in shoot length response (Fig. 3c).

Enset plantlets were transferred to sterile soil containing manure and acclimatized in a humid chamber in the glasshouse for 4 weeks (Fig. 1j). The potting mixture normally used is loamy soil and some other soft and water-retaining material (such as peat moss, vermiculite or red ash) mixed in a ratio of 1:1. Almost all the plants acclimatized in the glasshouse conditions, which is higher than the previous report of about 80% survival rate [19]. Successful acclimatization of plantlets largely depends on the sterility of the potting materials [20]. Acclimatized plantlets were transferred to bigger pots and grown under glasshouse conditions (Fig. 1k).

Organized cultures, especially the shoot tips, maintain strict genotypic and phenotypic stability under tissue culture conditions [25]. However, for enset, shoot tip culture resulted in high incidences (27%) of somaclonal variation (off-types). About 4–15% of the potted plants in the glasshouse showed wrinkled leaves, dwarfism or/and narrow leaves. Within genus *Musa*, shoot-tip cultures produced off-types at different rates ranging from 6 to 38% for Cavendish cultivars (*Musa* spp. AAA group) and 74% in plantain (*Musa* spp. AAB group) [39]. Evidently, the rate of somaclonal variation development is strongly influenced by genetic stability of each genotype, and its frequency is intensified by culture-induced factors. Tissue culture factors such as the number of subcultures [40] or the proliferation rates [39, 41] in vitro can strongly influence the incidence of somaclonal variation in the genus *Musa*. To reduce the incidence of somaclonal variation in enset cultivar Bedadeti, shoot tip explants for bud induction were routinely obtained from screen-house-grown plants rather than from plantlets maintained in vitro. Additionally, induced multiple buds were allowed to proliferate for a maximum of two subcultures of 14–21 days each.

### Sensitivity of multiple buds to kanamycin

**Fig. 4:**
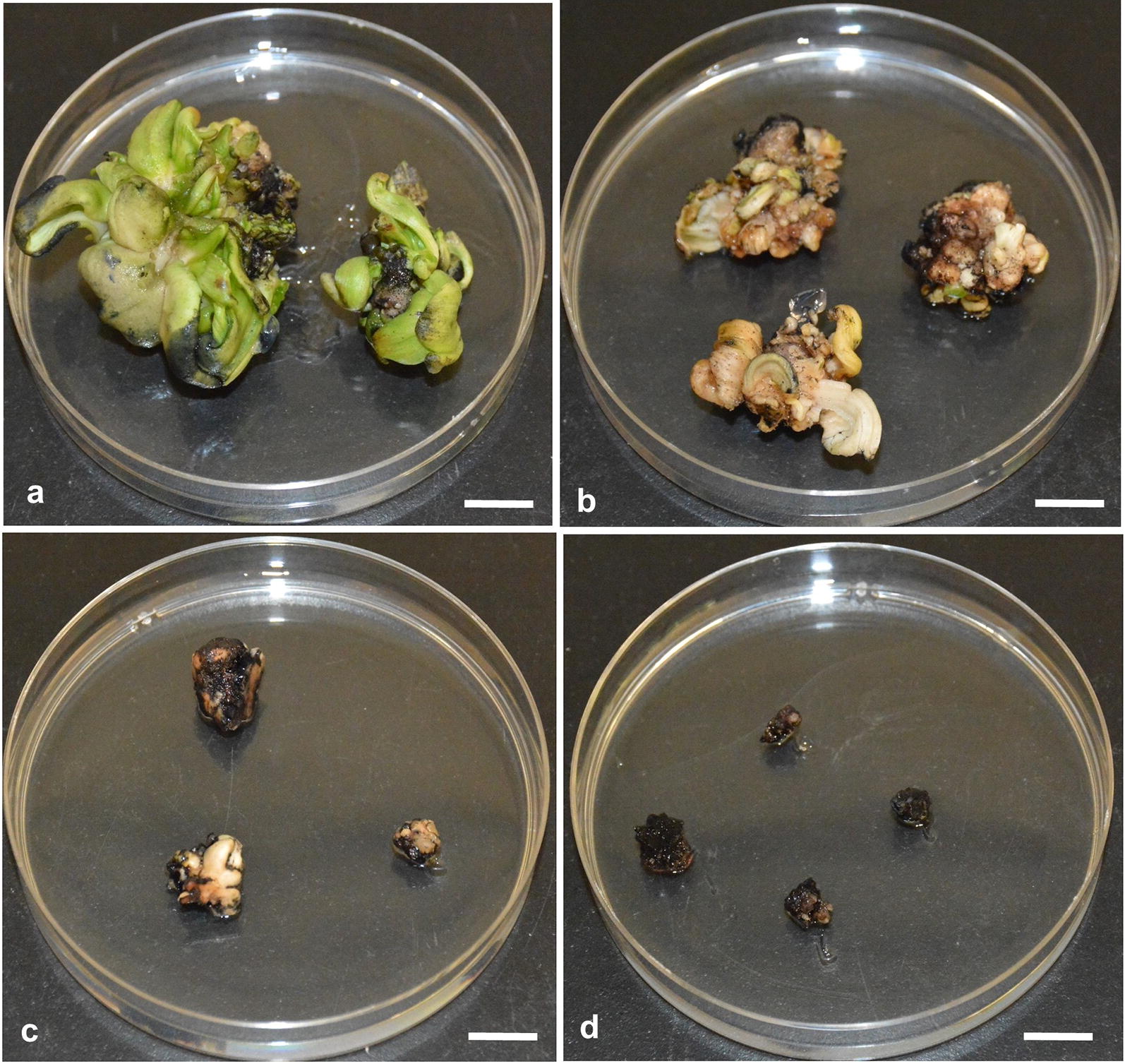
Effect of different concentration of kanamycin on shoot regeneration from multiple buds of enset cv. Bedadeti cultured on selective regeneration medium supplemented with kanamycin. **a** 50 mg/l, **b** 100 mg/l, **c** 150 mg/l, **d** 200 mg/l. Pictures were taken 60 days post culture of multiple buds on selective medium using a digital camera (Nikon camera Model D7100, Nikon Corporation, Tokyo, Japan). Scale bar = 1 cm

An effective selection strategy is very important to develop an efficient genetic transformation procedure. This can be achieved by the use of a selective agent which prevents non-transformed tissues from regenerating, while permitting the development of transformed cells into shoots without any lethality of the explant tissues [42]. The type of selectable marker genes and the selection pressure are very important factors for successful transformation. In this study, the *npt*II gene, which confers the resistance to kanamycin, was used based on its application on other *Musa* species including banana [8, 23] and plantain [43]. To determine the selection pressure suitable for enset, multiple bud explants were cultured on media supplemented with different concentrations of kanamycin (0, 50, 100, 150, 200 mg/l). Multiple bud explants started to display kanamycin stress symptoms on selective shoot induction media within 4 weeks of culture. Within 8 weeks of culture, explants cultured on 0 or 50 mg/l kanamycin grew healthily and induced buds, which sprouted into green shoots (Fig. 4a). After 8 weeks on media with 100 or 150 mg/l kanamycin, explants developed bleached buds (Fig. 4b, c). There was no bud or shoot development on media having 200 mg/l kanamycin (Fig. 4d). Therefore, 150 mg/l of kanamycin was chosen as the minimum inhibitory concentration for selection of transformed enset tissues and shoots.

### Optimization of enset transformation using multiple buds as explants

To develop a rapid protocol for generating transgenic enset plants using *A. tumefaciens*, the associated parameters were individually optimized using the established regeneration protocol described earlier. The parameters studied included the micro-wounding of explants by sonication prior to co-cultivation, *Agrobacterium* strain, co-cultivation medium and resting duration for the recovery of *Agro*-infected tissues prior to the application of antibiotic selection.

#### Effect of micro-wounding of explants on transformation

**Fig. 5:**
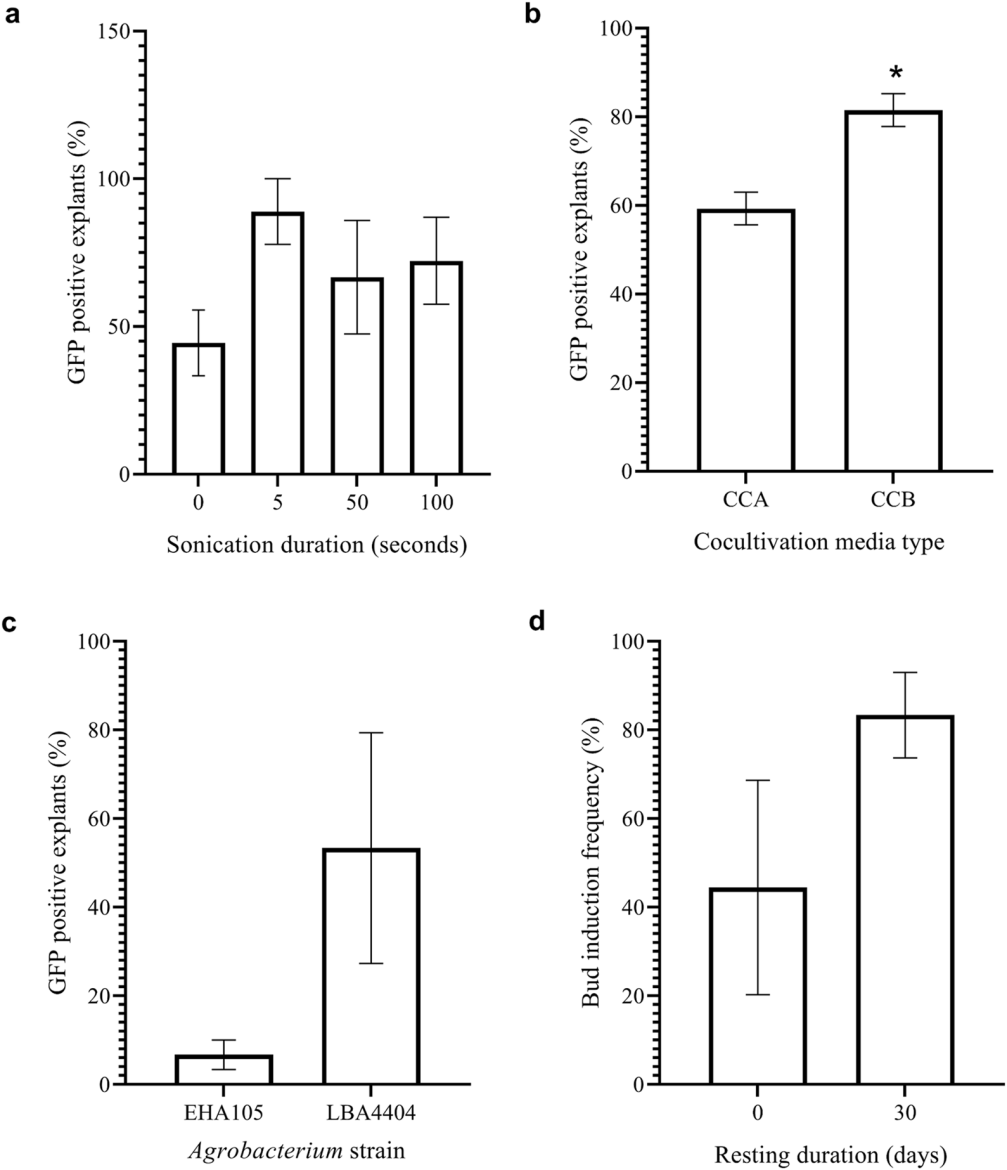
Optimization of different parameters for establishing transformation of enset using multiple buds. **a** Effect of sonication duration on transient expression of *gfp* reporter gene as percentage of multiple bud explants showing green fluorescence, **b** effect of types of cocultivation media on transient expression of *gfp* gene as percentage of multiple bud explants showing green fluorescence, **c** effect of *Agrobacterium* strains on transient expression of *gfp* gene as percentage of multiple bud explants showing green fluorescence, **d** effect of resting duration of *Agro*-infected multiple bud explants surviving on media with 150 mg/l kanamycin. The bars show the mean and standard error for values of expression of *gfp* gene as percentage of multiple bud explants showing green fluorescence and explant survival on selection media

The effect of micro-wounding of explants, through sonicating them for various durations, on transformation frequency was investigated. Multiple buds were sonicated for different times (0–100 s) and evaluated for transformation frequency following *Agrobacterium* mediated-transformation. Our results indicated that sonicated explants had higher transient expression of *gfp* gene in comparison to non-sonicated tissues (Fig. 5a). These results are in accordance with previous results that indicate that sonication of tissues enhances transformation [27]. Sonication-assisted *Agrobacterium*-mediated transformation (SAAT) causes increased secretion of phenolic compounds from the micro-wounded surface and sub-surface layers of targeted tissue. Increased production of phenolic compounds attracts more *Agrobacterium* cells to the site of wounding, leading to an enhancement in transformation efficiency [27]. Further, wounding of multiple buds has contributed to the generation of homogenously transformed plants [23, 24]. Our study showed that enset transformation was enhanced when explants were sonicated for 5 s resulting in a higher expression of *gfp* gene in comparison to other treatments (Fig. 5a). In addition, none of the treatments affected the regeneration efficiency of explants. For all subsequent transformation experiments, all explants were sonicated for 5 s prior to co-cultivation.

#### Effect of co-cultivation medium on transformation

The effect of different co-cultivation media (CCA medium without glucose and CCB medium with glucose) on transformation frequency was investigated. Co-cultivation of explants with *Agrobacterium* in the glucose-containing CCB medium resulted in a significantly higher (*p* = 0.0132) transformation efficiency than cultivation on CCA medium (Fig. 5b). However, the CCB media also promoted heavy overgrowth of *Agrobacterium* on explants. The explants cultured on the CCB medium could not be recovered by washing off excess *Agrobacterium* with RM media containing 500 mg/l cefotaxime and explants became highly blackened shortly after culturing on resting media. The enhancement of transformation frequency by CCB medium can be attributed to the presence of glucose. Glucose and other aldose monosaccharides activate the chromosomally encoded periplasmic protein (ChvE) which mediates a sugar-induced increase in *Vir* gene expression as well as *Agrobacterium* chemotaxis. In addition, glucose works synergistically with acetosyringone to enhance the transformation frequency [44]. Similar to transformation procedures for other monocots, acetosyringone (200 µM) was added to the co-culture media to induce *A. tumefaciens* to infect plants cells. Acetosyringone works by activating the *Vir* gene found on the *A. tumefaciens* helper plasmid. Our results showed less overgrowth of *Agrobacterium* when explants were co-cultivated in CCA medium. Therefore, CCA medium was used in all subsequent experiments.

#### Effect of *Agrobacterium* strain on transformation

The effect of *Agrobacterium* strain on transformation frequency was investigated. The transformation frequencies of enset multiple bud infection with the Agropine strain EHA105 or the Octopine strain LBA4404 of *A. tumefaciens* were compared. Results indicated that enset is sensitive to both EHA105 and LBA4404, however, LBA4404 produced higher transformation frequencies than EHA105 (Fig. 5c). One of the challenges associated with *A. tumefaciens*-mediated transformation is the variability in competency of different *Agrobacterium* strains to plant infection. This implies that susceptibility to *Agrobacterium* is species-specific, making it essential to identify the best strain for the crop under study. EHA105 has been used as the strain of choice in the transformation of different *Musa* species including banana [8, 45, 46] and plantain [47]. However, efficient transformation of enset was achieved using LBA4404 in our study. Consequently, LBA4404 was used in subsequent stable transformation experiments.

#### Effect of resting phase on transformation

The effect of resting phase without a selective agent on the regeneration of infected explants was evaluated. The regeneration response of *Agro*-infected explants to different resting durations (0 or 30 days) was compared. The transformation method involving a resting phase of 30 days resulted in sprouting of 84% of *A*gro-infected explants, whereas the method devoid of resting phase showed a regeneration of only 44% of explants (Fig. 5d). Several transformation protocols of banana and plantain recommend the culture of *Agro*-infected explants on non-selective media after co-cultivation for better transformation efficiency [48]. The resting phase allows explants to recover from the shock and damage associated with *Agro*-infection thereby increasing their chances of surviving on selective medium. In addition, the resting phase gives the opportunity to small and slow-responding cells to grow, thereby improving the tolerance of putative transgenic tissues to selective agent. This may result in better differentiation and sprouting in later stages of regeneration. Our results showed enhanced transformation of enset when *Agro*-infected explants were rested before selection. Consequently, a resting phase was included in all the subsequent transformation experiments.

#### Stable transformation and chimera dissociation in transgenic plants

**Fig. 6:**
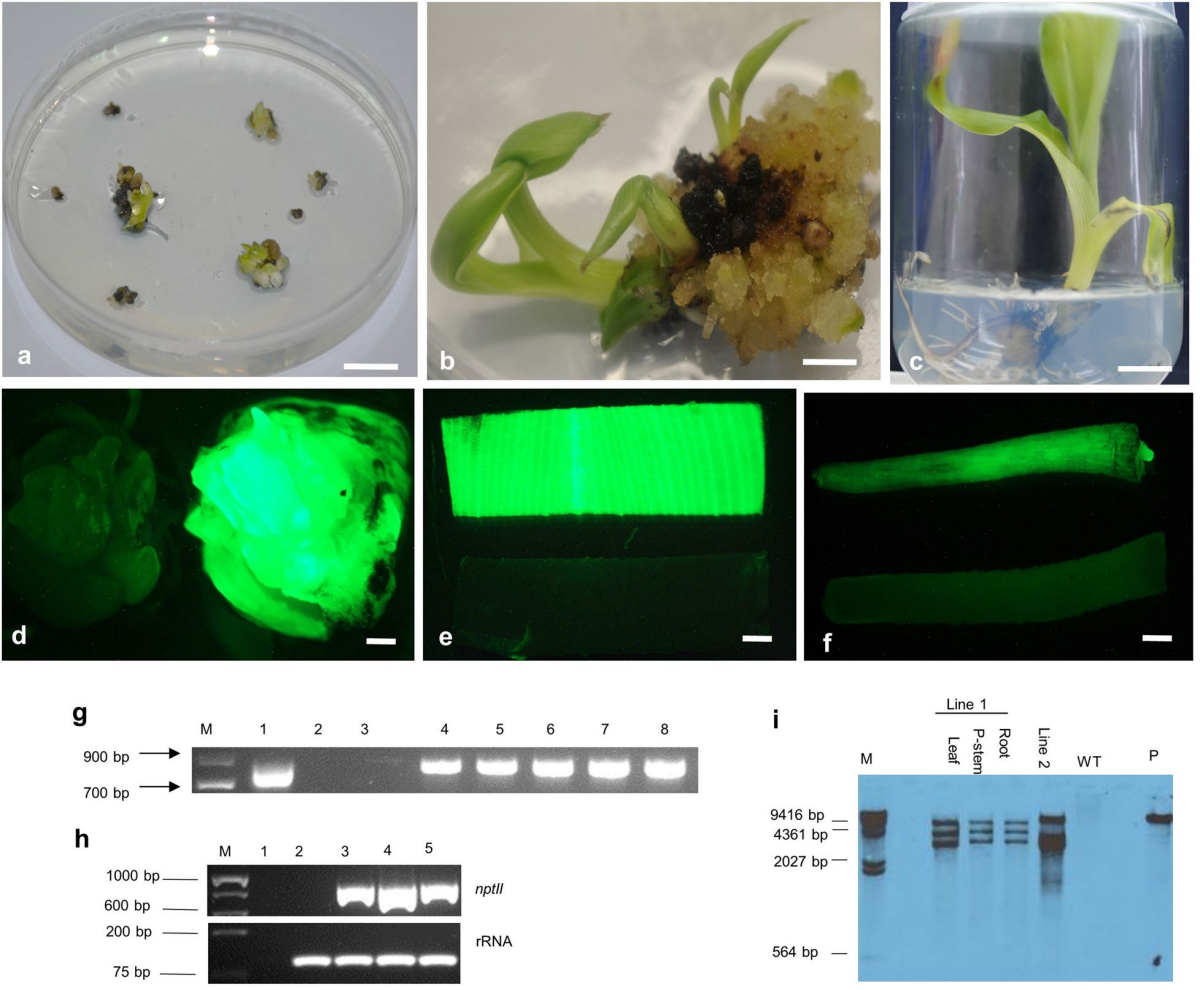
Generation and characterization of transgenic events of enset using *gfp* as reporter gene. **a** *Agro*-infected multiple bud explants cultured on selective medium 30 days after transformation, **b** regeneration of shoots from *Agro*-infected explants on selective medium, **c** fully developed transgenic plantlet after 5 cycles of selection for chimera dissociation on medium having 150 mg/l kanamycin, **d** distinct bud showing expression of g*fp* gene post 60 days after co-cultivation. **e**, **f** Expression of *gfp* gene in leaf and root tissues excised from transgenic plantlet, **g** PCR amplification of the *npt*II gene in different chimera diluted transgenic events. Lane M, 1 Kb plus DNA ladder; Lane 1, pCAMBIA2300-GFP; Lane 2, non-template control; Lane 3, non-transformed control; Lane 4–8, chimera diluted transgenic events 1–5, **h** RT-PCR analysis of different tissues of the uniformly transformed plantlets using primers specific to *npt*II gene. Lane M, 100 bp ladder; Lane 1, non-template control; Lane 2, non-transformed control; Lane 3, Leaf of transgenic plantlet; Lane 4, pseudostem of transgenic plantlet; Lane 5, root of transgenic plantlet. **i** Southern hybridization analysis of transformed plantlets. Genomic DNA was digested with HindIII and probed with a DIG-labelled fragment (780 bp) of *npt*II gene. Lane M, DIG-labelled Lambda HindIII ladder; Lane WT, non-transformed control DNA; Lane P, pCAMBIA2300-GFP, Scale bar = 1 cm in **a**–**c** and 0.5 cm in **d**–**f**

Stable transformation of enset was achieved using the best responses for the parameters tested. This involved infection of micro-wounded explants with *Agrobacterium* strain LBA4404 using co-cultivation medium CCA for 3 days. *Agro*-infected explants were cultured on medium without selection for 30 days before regeneration of explants on SRM containing 150 mg/l kanamycin. Shoot induction occurred within 6–8 weeks with the explants producing distinct buds on SRM (Fig. 6a; Additional file 2: Fig. S2a). After 8–9 weeks of selection, the buds sprouted into shoots (Fig. 6b). About 11.9% of the *Agro*-infected explants showed regeneration on SRM, however, only 4.33% *Agro*-infected explants developed putative transgenic shoots. A total of 12 putative transformed shoots were obtained, which were validated by PCR analysis using primers specific to *npt*II gene. Eight out of 12 transgenic events were PCR positive, confirming the presence of *npt*II gene. UV illumination from a fluorescence stereomicroscope also detected stable expression of *gfp* gene in buds and regenerated tissues of these transformants (Fig. 6d–f). To ensure that all plantlets were uniformly transformed, the PCR positive transgenic events were subjected to chimera dissociation.

Very high incidences of chimeric plantlets (over 60%) have been reported for meristem transformation in different monocot species including banana [23], maize [49], rice [50] and bluegrass [51]. One way of overcoming this technological barrier for *Musa* species may be to perform a second round of selection for all putative transformants [8, 23]. Therefore, in this study, all regenerated transgenic events underwent chimera dissociation treatment consisting of the culture of shoot tip explants on CDM1 for 7 days followed by the culture on CDM2 for 14 days. Several studies reported that two cycles of explant regeneration on high kanamycin (100–150 mg/l) was sufficient for successful chimera dissociation [8, 23, 26]. However, development of a robust root system from explants on selective medium may be a more reliable indicator of uniform transformation [22, 23]. Therefore, in this study, the emergence of roots on explants exposed to 150 mg/l kanamycin was used as an indicator of successful dissociation of chimeras. Our results indicated that the majority of the explants survived (with some rooting) on cycle 1 of chimera dissociation. However, most (over 60%) of the explants did not grow on cycle 2 and 3, but instead turned brown and died or produced bleached shoots (Additional file 2: Fig. S2b). The surviving green shoots were transferred to 4th cycle of selection (Additional file 2: Fig. S2c). Roots were initiated when the surviving shoots were transferred to the 4th cycle of selection. A final (5th) cycle of selection was therefore necessary to achieve full plantlet development. After 5 cycles of selection, the fully developed shoots were deep green in color and well rooted (Fig. 6c). Five out of the 8 transgenic events survived chimera dilution. These surviving events were confirmed uniformly transformed through PCR, RT-PCR and Southern blot hybridization. Surviving plantlets were maintained on SEM media (Additional file 2: Fig. S2d).

#### Molecular analyses of transgenic events

The transgenic events that survived extensive selection for chimera dilution were characterized by PCR using *npt*II gene-specific primers to confirm the presence of transgene. The expected fragment size of 780 bp was obtained in the transgenic plants (Fig. 6g, lanes 4–8) and positive control pCAMBIA2300-GFP plasmid DNA (Fig. 6g, lane 1). No amplicons were observed either on the non-template control (Fig. 6g, lane 2) or in the DNA from the non-transformed plantlets (Fig. 6g, lane 3).

Transgene expression was analyzed by RT-PCR using *npt*II gene-specific primers and cDNA prepared from different tissues of the chimera dissociated transgenic plant. The results revealed the expression of the *npt*II gene in all tissues of the transgenic plant but not in the untransformed control plant (Fig. 6h). A transformation efficiency of 1.25% was achieved on the basis of the number of *Agro*-infected explants compared with the number of uniformly transformed PCR positive plants.

To further confirm the integration of transgene, Southern blot analysis was performed on gDNA obtained from two chimera-diluted events using the fragment of *npt*II gene as the probe. Figure 6i shows *npt*II hybridizing signals of the expected sizes for the control pCAMBIA2300-GFP plasmid (11,634 bp) and two transgenic events. There was consistent *npt*II hybridizing signals in leaf, pseudostem and root samples of the transgenic line 1. The Southern hybridization results also indicate that the T-DNA integrated into three sites on the genome of the transgenic line 1 and 2. However the integration pattern for the two lines was different. No hybridization signal was detected in the non-transformed control (Fig. 6i).

**Fig. 7:**
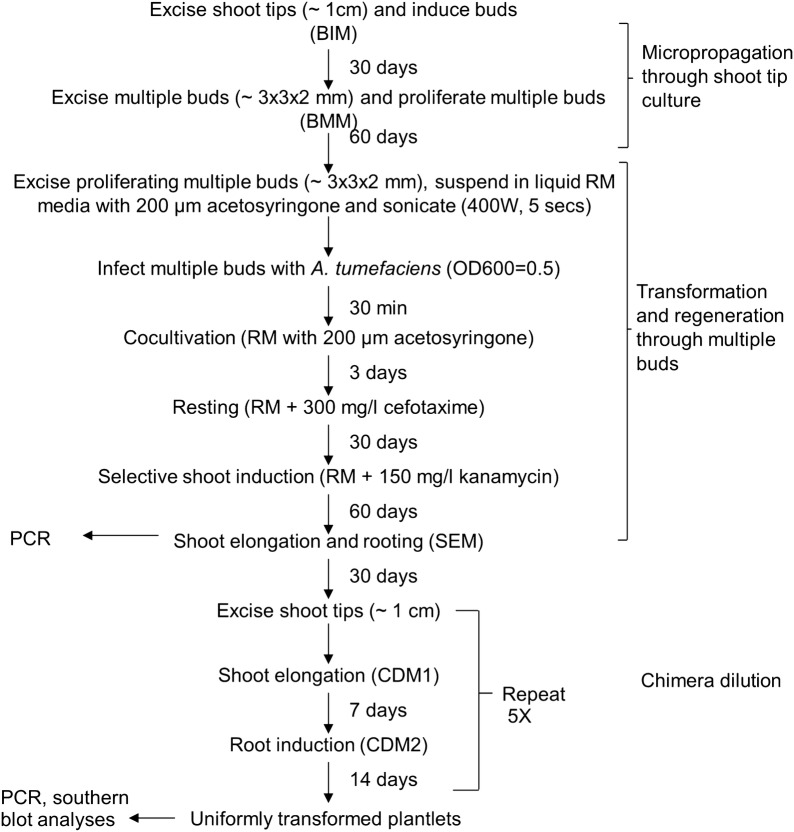
Schematic flow diagram showing steps in genetic transformation of enset using proliferating multiple buds as explants. BIM, bud induction medium; BMM, bud multiplication medium; RM, regeneration medium; SEM, shoot elongation medium; CDM1, chimera dilution medium 1; CDM2, chimera dilution medium 2

The molecular analysis revealed a consistent transgene integration and expression pattern across all tissues of the transgenic plant, implying stable and uniform transformation of enset. Using the optimized protocol, putative transgenic enset plants can be generated in 4 months. A further 4 months is required for chimera dissociation to obtain uniformly transformed plantlets (Fig. 7).

## Conclusion

This study reports a protocol for *Agrobacterium*-mediated transformation and regeneration of *E. ventricosum*. To our knowledge, this is the first report on stable transformation and regeneration of transgenic enset plants. The system, which is based on regeneration through multiple buds, is simple and delivers transgenic plants at an efficiency of 1.25%. This protocol has been tested on Bedadeti and other cultivars in the laboratory. The study offers a method for genetic improvement of *E. ventricosum* cultivars and other *Ensete* species. Work is in progress to develop bacterial wilt-resistant *E. ventricosum* using the transformation protocol developed in this study.

## Supplementary information

**Additional file 1: Fig. S1.** Schematic representation of the T-DNA region of binary plasmid pCAMBIA2300-GFP. Primer binding regions for amplification of *npt*II gene are indicated.

**Additional file 2: Fig. S2.** Generation of uniformly transformed transgenic events of enset. (a) *Agro*-infected explants cultured on selective regeneration medium containing 150 mg/l kanamycin, (b) regeneration of shoot tips isolated from putative transgenic shoots at cycle 2 of chimera dilution on selective regeneration medium. Green shoots were further transferred to selective regeneration medium and bleached non-transgenic shoots were discarded, (c) putative transgenic shoots at Cycle 4 of chimera dilution on selective regeneration medium containing 150 mg/l kanamycin, (d) uniformly transformed plants maintained on SEM media with 2 mg/l BAP and 0.2% activated charcoal. Scale bar = 1.5 cm.

## Data Availability

All data generated or analyzed during this study are available in this published article.
