# Supplementary figures and images for: A simple and rapid protocol for the genetic transformation of Ensete ventricosum
Source: Plant Methods. 2019 Nov 8;15:130. doi: 10.1186/s13007-019-0512-y (PMC6839154; doi:10.1186/s13007-019-0512-y)

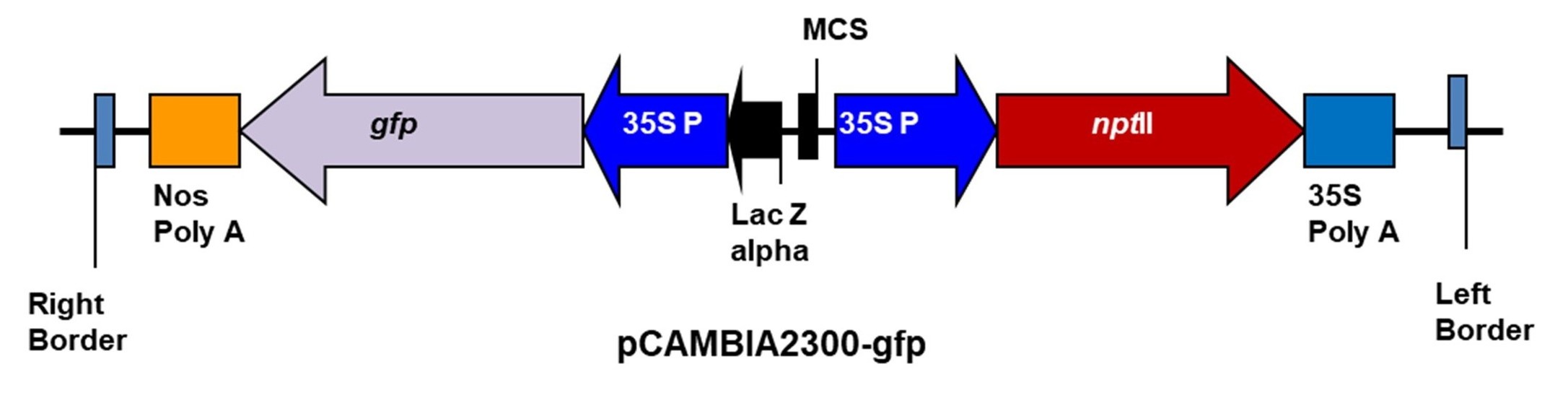

Supplement: Supplementary file 1 — Additional file 1: Fig. S1. Schematic representation of the T-DNA region of binary plasmid pCAMBIA2300-GFP. Primer binding regions for amplification of nptII gene are indicated. [file 13007_2019_512_MOESM1_ESM.jpg]

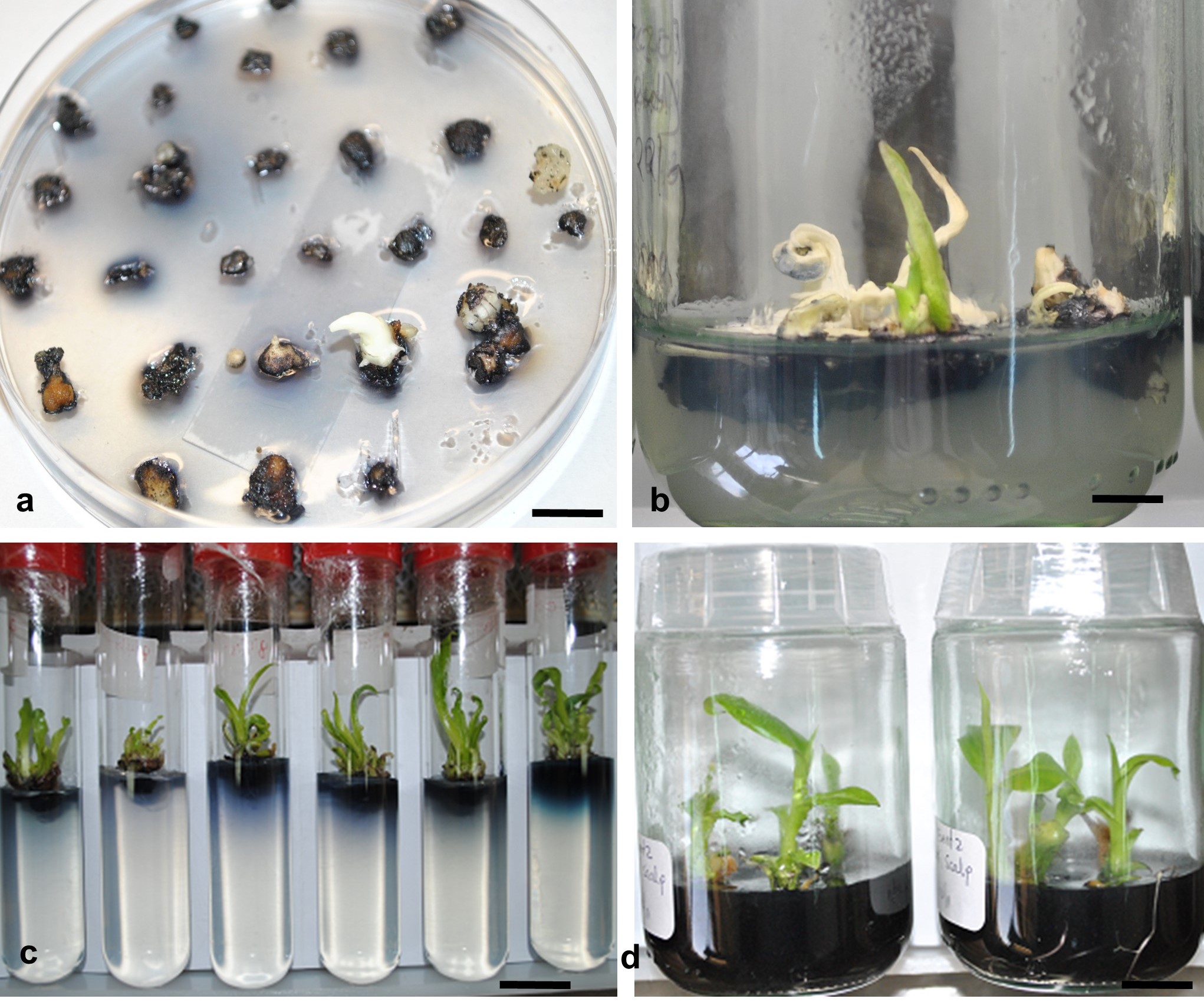

Supplement: Supplementary file 2 — Additional file 2: Fig. S2. Generation of uniformly transformed transgenic events of enset. (a) Agro-infected explants cultured on selective regeneration medium containing 150 mg/l kanamycin, (b) regeneration of shoot tips isolated from putative transgenic shoots at cycle 2 of chimera dilution on selective regeneration medium. Green shoots were further transferred to selective regeneration medium and bleached non-transgenic shoots were discarded, (c) putative transgenic shoots at Cycle 4 of chimera dilution on selective regeneration medium containing 150 mg/l kanamycin, (d) uniformly transformed plants maintained on SEM media with 2 mg/l BAP and 0.2% activated charcoal. Scale bar = 1.5 cm. [file 13007_2019_512_MOESM2_ESM.jpg]
